# Influence of Synthesis Method on LTA Time-Dependent Stability

**DOI:** 10.3390/molecules23092122

**Published:** 2018-08-23

**Authors:** Claudia Belviso, Antonio Lettino, Francesco Cavalcante

**Affiliations:** 1 Istituto di Metodologie per l’Analisi Ambientale—IMAA-CNR, 85050 Tito Scalo, Italy; antonio.lettino@imaa.cnr.it (A.L.); franceso.cavalcante@imaa.cnr.it (F.C.); 2 Istituto di Struttura della Materia—ISM-CNR, 85050 Tito Scalo, Italy

**Keywords:** LTA, zeolite, ultrasonic irradiation, hydrothermal method, stability

## Abstract

Time-stability of LTA zeolite formed by hydrothermal method with or without the action of ultrasonic irradiation was investigated by X-ray diffraction analysis (XRD) and scanning electron microscopy (SEM). The results show that 6 months after the synthesis by hydrothermal process with continuous sonication, LTA evolves into a more stable sodalite, whereas no differences are detected 12 months after LTA synthesis by conventional pre-fused hydrothermal process. These data confirm that using the two approaches, different mechanisms control both zeolite crystallization and time-stability of the newly-formed mineral at solid state. The results are particularly important in the light of the synthetic zeolite application.

## 1. Introduction

There are many literature data documenting the synthesis of zeolite using both processes requiring organic compounds as structure-directing agents [1,2,3,4,5] and template-free synthesis with aluminate and silicate solutions [5,6,7,8,9,10,11]. However, all these processes have been performed through various methods, among which hydrothermal treatment [12,13,14,15,16,17,18,19,20,21,22] and ultrasonic irradiation [23,24,25,26,27,28,29,30] have been widely used.

The synthesis of zeolite generally takes place in clear solution or in dispersed low-density sol-gel or in viscous gel [31], although some authors demonstrated that zeolite formation can also be performed by solvent-free process (solid phase) [32,33,34]. The mechanistic pathways in zeolite formation can be summarized in the following subsequent stages: Induction period, nucleation and crystal growth [13]. The induction period is the time between the speculative start of the reaction and the point at which crystalline structure is first observed. As far as the precipitation reaction is concerned, Cundy and Cox [13] indicated that the induction period is divided into three subunits, according to the classical nucleation theory [35]. The first is the time required by the starting system to achieve the equilibration reactions; the second and the third are, respectively, the time for the formation of stable nucleus and the time for the nucleus to grow to a detectable size. The nucleation can be expressed as the transformation from random structure into regular and periodic crystal lattice [13]. The kinetic of nucleation is the critical factor directly responsible for the product of zeolite synthesis. Ng et al. [36], in the introduction of their paper, indicated that this parameter is particularly important when the target is a zeolite structure with high free energy because this zeolite could be transformed into a more favorable phase from an energetic point of view. Zeolites are, in fact, metastable crystalline aluminosilicate, and their conversion to more-stable phases has often been observed. The final stage of zeolite mechanism formation is represented by the growth of the initial microcrystal into a well-defined zeolite product (crystal growth stage). Numerous parameters influence zeolite nucleation and growth, thus controlling not only the framework type but also the physicochemical properties of the final synthetic crystalline phase [37,38,39]. For zeolite A, indicated as LTA by the International Zeolite Association code [40], different hypothesis of nucleation mechanism and growth have been proposed [41,42] and even more numerous studies have been performed to investigate the subsequent collapse of this zeolite into hydroxosodalite, the more thermodynamically stable phase [43,44,45]. 

In our recent paper [46], the synthesis of zeolite A by both ultrasonic and conventional hydrothermal process was investigated. Fly ash was used as raw material. The comparison between the two methods indicated that ultrasonic energy is decisive in very fast zeolite transformation into more stable sodalite phase. In order to confirm these results and further investigate the influence of sonication during the hydrothermal process on LTA synthesis mechanism and time-stability, regardless of the raw material used, in this work new experiments with Na_2_O-Al_2_O_3_-SiO_2_-H_2_O precursor system were performed using both approaches. 

## 2. Results

**Figure 1:**
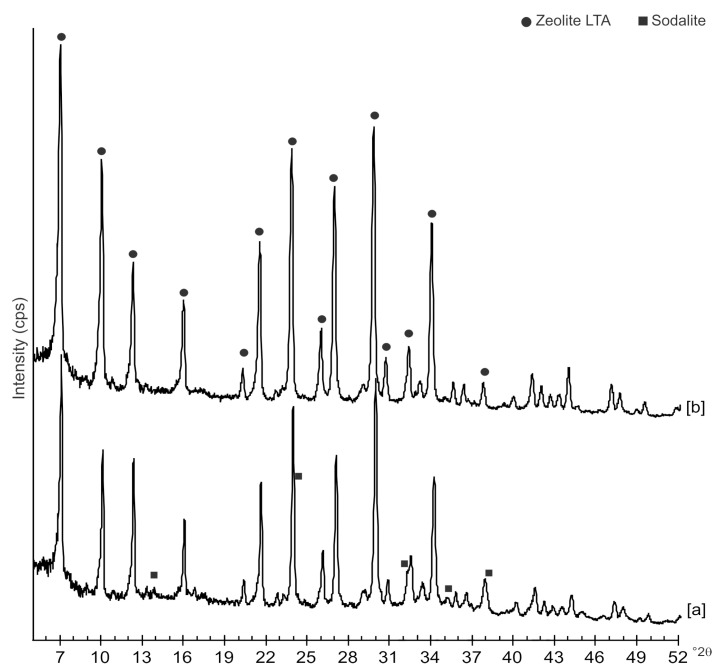
XRD patterns of samples synthesized after 4 day of: [a] hydrothermal process with ultrasonic irradiation and [b] conventional hydrothermal treatment.

The diffraction patterns of samples immediately after hydrothermal treatment with and without ultrasonic are shown in Figure 1. The peaks observed on both profiles are indexed to LTA zeolite. A minor impurity phase represented by Al oxide is also detected in both samples. A closer examination of XRD profiles from sample after sonication also reveals a minor phase indexed to sodalite structure.

**Figure 2:**
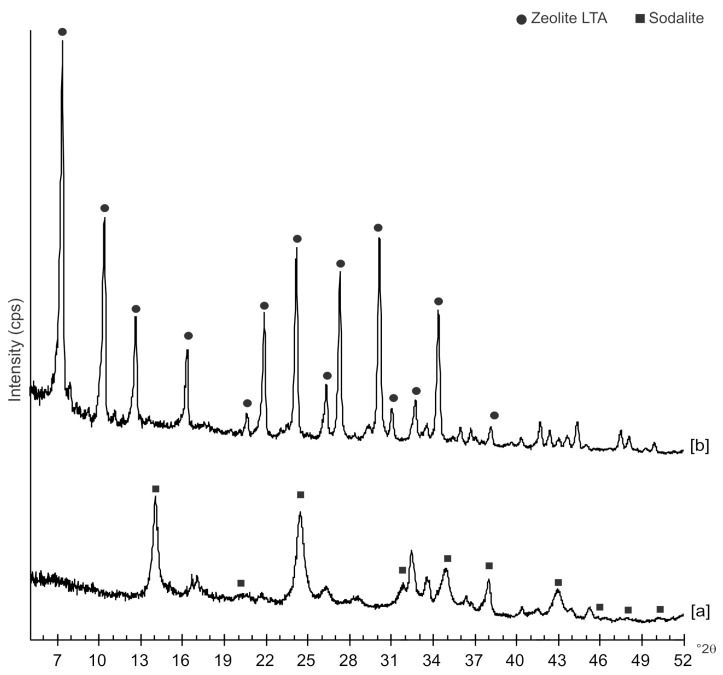
XRD profiles of sample analyzed: [a] 6 months after the synthesis by hydrothermal process with ultrasonic irradiation and [b] 12 months after the synthesis by conventional hydrothermal treatment.

Figure 2 displays the X-ray diffraction results of the investigation performed on the same samples but a few months after their synthesis. The data indicate that US sample, analyzed after 6 months, contains mainly sodalite, whereas this phase is not detected on XRD pattern of HY sample investigated 12 months after its formation. This sample is only characterized by the presence of LTA zeolite.

**Figure 3:**
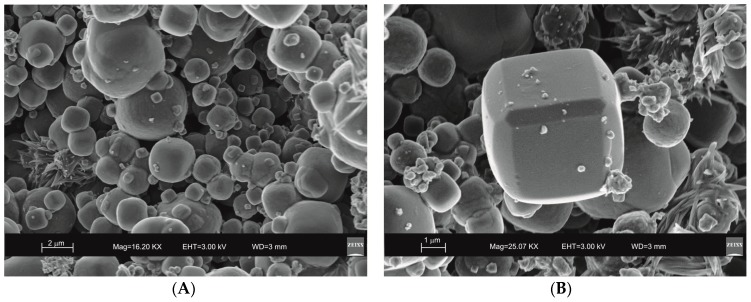
Scanning electron microscopy (SEM) images of LTA zeolite formed through 45 min of hydrothermal process with sonication. (**A**) overview of zeolites formed; (**B**) detail of LTA crystal.

**Figure 4:**
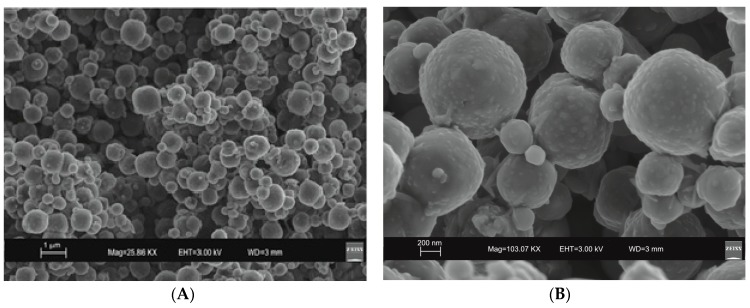
SEM images of LTA zeolite formed by a 4-day conventional hydrothermal incubation. (**A**) overview of zeolites formed; (**B**) zoom in on LTA crystals.

SEM images reveal the typical crystalline cubic morphology of zeolite LTA formed after 45 min of US treatment and after 4 days of HY process as shown in Figure 3 and Figure 4, respectively.

**Figure 5:**
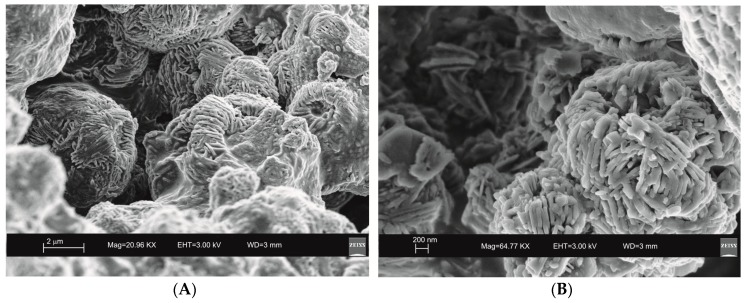
SEM images of sodaline characterizing the sample 6 months after the ultrasonic water bath treatment. (**A**) overview of zeolites formed; (**B**) detail of sodalite crystal.

**Figure 6:**
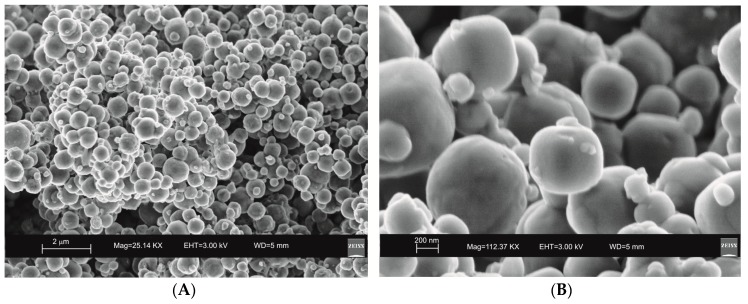
SEM images of LTA zeolite characterizing the sample 12 months after the conventional hydrothermal treatment. (**A**) overview of zeolites formed; (**B**) zoom in on LTA crystals.

The analysis performed on the solid samples 6 and 12 months after their synthesis by sonication and hydrothermal incubation confirms the presence of sodalite (Figure 5) and LTA zeolite (Figure 6), respectively.

## 3. Discussion

The results indicate that LTA formed by both hydrothermal process with ultrasonic irradiation and, conventional hydrothermal treatment although well definite crystals of this zeolite synthesized at a shorter time (45 min) with US method, thus confirming literature data [47]. 

**Figure 7:**
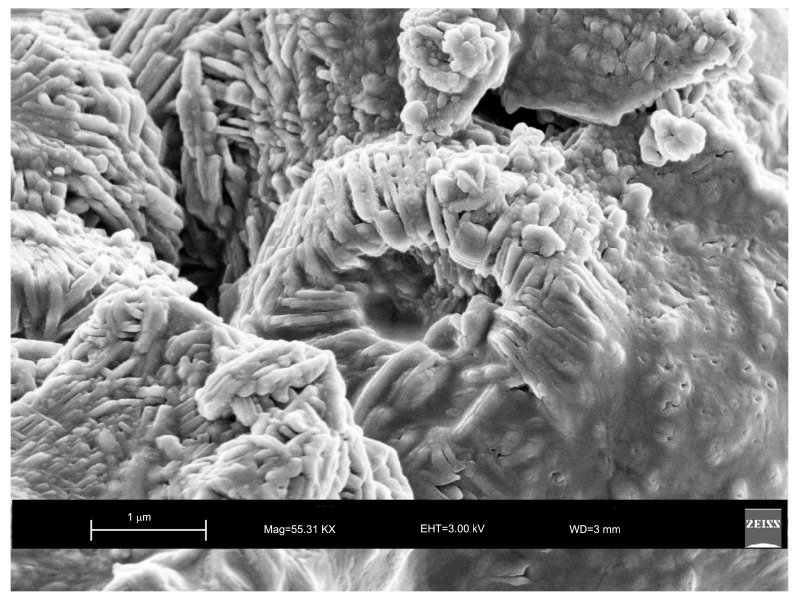
SEM image showing well defined plate morphology of sodalite crystal formed 6 months after hydrothermal treatment with ultrasonic.

Moreover, the US pattern shows sharper diffraction peaks of zeolite (Figure 1) as displayed by the peak widths at half-height of the peaks (FWHM) at about 7° 2θ (200) which are 0.19° Δ2θ for the US sample and 0.57° Δ2θ for the HY sample. This is indicative of a larger crystal dimension of the newly-formed mineral synthesized by sonication process. The results are confirmed by the morphological study performed by SEM. Figure 3, in fact, shows that zeolite crystals formed by sonication are bigger and the edges of the typical cubic morphology are well defined when compared to the cubic morphology of the same zeolite formed by hydrothermal process (Figure 4). These data do not seem to be in accordance with our previous results about the size of zeolite formed using sonication [29,30]. However, a more careful examination reveals that the results are not contradictory. In previous studies [29,30], in fact, we discussed the action of ultrasonic being applied before the conventional hydrothermal process and we demonstrated the ability of sonication in Al-Si enrichment as well as in the increase of nuclei to be involved into the nucleation rate of crystalline phases during the following step of hydrothermal incubation. The application of hydrothermal treatment with the continuous action of sonication as performed in this study, in contrast, due to the cavitation bubble [48], increased the number of nuclei in the system, thus promoting their aggregation and following formation of large LTA crystals [47] (Figure 1 and Figure 3). The large particle size indicates a decreased induction time and an increased crystal growth rate, in accordance with literature data [47]. However, LTA zeolite formed in a short time (45 min) by ultrasonic irradiation shows a low stability over time. A fast evolution to more stable sodalite was, in fact, detected at environmental conditions in spite of literature data, indicating that LTA to sodalite solid state transformation is nearly impossible due to the large amount of energy required [25,45]. X-ray diffraction analysis, in contrast, displays the main presence of sodalite on the XRD pattern of the samples analyzed after the 6-month synthesis (Figure 2), whereas SEM images show the presence of the typical rose-like morphology of sodalite crystals (Figure 5). Our hypothesis is that the large aggregate formed by US treatment represents a crucial step also for the LTA to sodalite transformation. According to the mechanism explained by Greer et al. [45], in fact, the nucleation of sodalite having a higher density in comparison with zeolite A takes place over time involving the amorphous material still characterizing the sample after the LTA zeolite formation. In particular, during this process the amorphous core of cubic LTA particles is probably involved. This is where the pressure is built up [45]. On the basis of the study performed by Greer et al. [45], sodalite nanoplates expand in size to break through the zeolite A crystalline morphology, and then the consumption of LTA occurs through an Ostwald ripening process [49]. A detailed analysis of SEM pictures confirms this mechanism. Figure 7 shows how well defined the plates of sodalite morphology are inside the core of relict crystal and their growth takes place from the inside outward. On the lower right side of the picture, characterized by the presence of amorphous material, the plate morphology clearly begins to emerge from below the surface of the amorphous phase.

**Figure 8:**
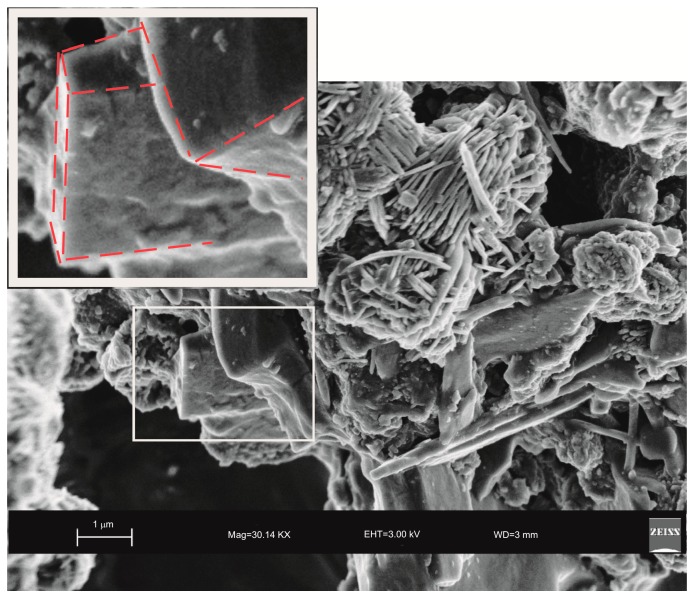
SEM image showing cubic face morphology of relict LTA zeolite (highlighted and enlarged part) detectable between sodalite crystals.

In Figure 8, in contrast, the cubic face morphology of relict zeolite is still detectable just because the consumption of LTA is not completed.

**Figure 9:**
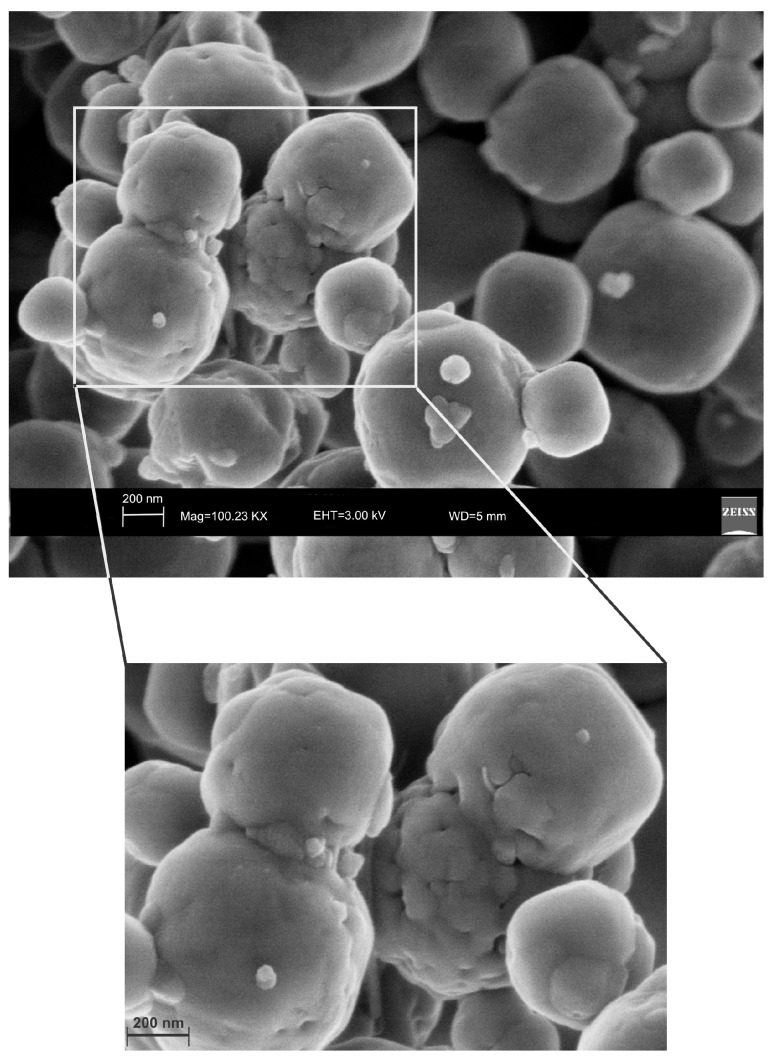
SEM images of sample 12 months after the synthesis by HY. They show isolated LTA crystal affected by an initial process of transformation into sodalite.

The mechanism controlling LTA zeolite synthesis by the conventional hydrothermal process is marked by slow and successive stages starting from the geopolymers/amorphous material formation with some zeolite nuclei from saturated solution and following small crystals nucleation when the chemical composition of the amorphous material is close to the stechiometric LTA composition. Finally, the slow LTA crystal growth both within the amorphous mass and at the liquid–solid interface takes place [15]. As previously discussed, both XRD and SEM data (Figure 1 and Figure 4) indicate that the newly-formed minerals are smaller in size if compared to the LTA synthesized by US treatment. This data excludes the nuclei aggregation as the main directing agent for the zeolite crystallization. Moreover, the absence of sodalite peaks on XRD pattern after 12 months (Figure 2 and Figure 6) indicates that the solid-state transformation of metastable zeolite into a more stable sodalite phase takes place through a very slow action involving LTA zeolite crystals without excluding the amorphous phase. A detailed morphological analysis performed on the sample 12 months after the synthesis by HY confirms this hypothesis. Figure 9, in fact, shows the SEM image of isolated LTA crystal affected by an initial process of transformation into sodalite.

Although the data indicate that two different mechanisms control both LTA crystallization and its time-stability, some trace on X-ray pattern of the samples formed by US treatment (Figure 1) as well as the presence of small and not well organized sodalite crystals in SEM picture (Figure 3) could not totally rule out the mechanism of precursors/nuclei for the synthesis in the conventional hydrothermal process. To confirm our XRD and SEM data and further study the solid-state zeolite evolution with respect to time, different technical methods such as differential scanning calorimetry (DSC) and transmission electron microscopy (TEM) will be used in our future study.

## 4. Experimental Section

### 4.1. Synthesis of LTA Zeolite

LTA zeolite was synthesized as follows: initially 80 mL of distilled water and 0.723 g of sodium hydroxide were mixed gently until NaOH completely dissolved (Solution 1). Then solution A was prepared by dissolving 15.48 g of sodium silicate in one-half of Solution 1. Solution B was prepared dissolving 8.258 g of sodium aluminate in the second half of Solution 1. Finally, Solution A was added quickly into Solution B under vigorous stirring to give a gel mixture with the following chemical composition: 3.165 Na_2_O:Al_2_O_3_:1.926 SiO_2_:128H_2_O. One half of the gel was subject to 45 min of treatment in ultrasonic water bath (US) (240 W; 35 kHz) and the other half was incubated in a conventional hydrothermal water bath (HY) for 4 days. The temperature of both treatments was comparable and fixed around 40 °C. After both US and HY processes, the solids and solutions were separated by centrifugation at 4500 rpm for 15 min. The solids were washed with distilled water, dried in an oven for 12 h at 80 °C and immediately analyzed. Then the samples were stored at ambient temperature and atmospheric condition away from light and analyzed again after 6 and 12 months. All chemicals and solvents (Aldrich Chemicals Ltd., Milan, Italy) were of reagent grade.

### 4.2. Characterization of Synthetic Products

The mineralogical composition of the samples was determined by X-ray powder diffraction (XRD) using a powder diffractometer (Rigaku Rint Miniflex, Tokyo, Japan) with Cu-Kα radiation; sample spinner and 30 kV × 15 mA. The size and morphology of the crystals were analyzed by a scanning electron microscopy (Zeiss Supra 40 SEM, Jena, Germany) equipped with an energy dispersive spectrometer (EDS). The synthetic products were examined immediately after the synthesis and also after a few months of aging.

## 5. Conclusions

The data show that zeolite crystallization by both hydrothermal processes with and without continuous ultrasonic irradiation is controlled by two distinct mechanisms also conditioning time-stability of newly-formed minerals. The results indicate that the quick LTA crystallization by sonication treatment ensures a fast transformation into the more stable sodalite. The slower mechanism of geopolymer transformation into crystalline phases by the 2-step conventional hydrothermal process, instead, is responsible for a very slow transformation of LTA into sodalite (detectable only after 12 months). These conclusions are based on XRD and SEM measurements, thus demonstrating the effectiveness of conventional laboratory techniques in determining the complex mechanism of zeolite crystallization and time-stability. However, mainly regarding the study of the time-dependent stability of the newly formed zeolite, more sophisticated analysis is planned in the future to confirm the understanding of the mechanism.

The time-dependent stability of synthetic zeolites represents an important parameter in the light of the storage and application of these minerals. It is well known that zeolites are very useful in many contexts ranging from environmental remediation [50,51] to catalysis [52]. However, to our knowledge, no study has investigated the potential solid-state transformation of zeolites during their long-time use as well as the consequent potential changes in their efficiency. The method to be used for the zeolite synthesis could therefore be decisive depending on the context of the application of these minerals.
